# Insertion Sequence Inversions Mediated by Ectopic Recombination between Terminal Inverted Repeats

**DOI:** 10.1371/journal.pone.0015654

**Published:** 2010-12-20

**Authors:** Alison Ling, Richard Cordaux

**Affiliations:** Université de Poitiers, CNRS UMR 6556 Ecologie, Evolution, Symbiose, Poitiers, France; Louisiana State University, United States of America

## Abstract

Transposable elements are widely distributed and diverse in both eukaryotes and prokaryotes, as exemplified by DNA transposons. As a result, they represent a considerable source of genomic variation, for example through ectopic (i.e. non-allelic homologous) recombination events between transposable element copies, resulting in genomic rearrangements. Ectopic recombination may also take place between homologous sequences located *within* transposable element sequences. DNA transposons are typically bounded by terminal inverted repeats (TIRs). Ectopic recombination between TIRs is expected to result in DNA transposon inversions. However, such inversions have barely been documented. In this study, we report natural inversions of the most common prokaryotic DNA transposons: insertion sequences (IS). We identified natural TIR-TIR recombination-mediated inversions in 9% of IS insertion loci investigated in *Wolbachia* bacteria, which suggests that recombination between IS TIRs may be a quite common, albeit largely overlooked, source of genomic diversity in bacteria. We suggest that inversions may impede IS survival and proliferation in the host genome by altering transpositional activity. They may also alter genomic instability by modulating the outcome of ectopic recombination events between IS copies in various orientations. This study represents the first report of TIR-TIR recombination within bacterial IS elements and it thereby uncovers a novel mechanism of structural variation for this class of prokaryotic transposable elements.

## Introduction

Transposable elements are discrete pieces of DNA that can move from site to site within (and sometimes, between) genomes. They are widely distributed in both eukaryotes and prokaryotes. Because of their large distribution and extensive diversity, they represent a considerable source of genomic variation and as such, they constitute powerful drivers of genome evolution [1], [2]. The proliferation of transposable element copies in a genome generates numerous homologous sequences at various genomic sites. As a result, recombination often occurs between non-allelic homologous transposable element sequences (also known as ectopic recombination), leading to genomic rearrangements such as deletions, duplications and inversions. This process, which has been widely documented in both eukaryotes and prokaryotes, illustrates the deep impact transposable elements may have on genomic structural variation and instability [1]–[3].

Ectopic recombination is not restricted to interactions *between* transposable element sequences. It may also occur between homologous sequences located *within* transposable element sequences. Indeed, various classes of transposable elements carry repeated sequences at their boundaries, which are important for element mobility. For example, eukaryotic genomes often contain retrotransposons and/or endogenous retroviruses that are bounded by long terminal repeats (LTRs). LTRs are up to 5 kb-long sequences that are directly repeated at the 5′ and 3′ ends of these elements [4]. The two LTRs of single transposable element copies are prone to ectopic recombination, which results in the deletion of the intervening sequence [5]. Thus, during ectopic recombination between LTRs, a full-length LTR retrotransposon or endogenous retrovirus is replaced by a single LTR, termed solo-LTR. LTR-LTR recombination is an important source of genomic variation and evolution, as ∼85% of endogenous retroviruses are found as solo-LTRs in the human genome [6] and solo-LTR formation has been proposed to be a mechanism contributing to mitigating the increase in genome size caused by new transposable element insertions in plants [7].

DNA transposons represent another evolutionary successful class of transposable elements found in both eukaryotes and prokaryotes [8], [9]. DNA transposons are typically bounded by terminal inverted repeats (TIRs). TIRs are sequences ranging in size from a few bp to several kb that are repeated in opposite orientation at the 5′ and 3′ ends of these elements [4], [8], [9]. Isolated TIRs (or solo-TIRs) have been reported in various prokaryotic genomes [10]–[12]. However, their origin is unclear as ectopic recombination between TIRs of single copies is not expected to result in solo-TIRs (as for LTR elements), but in transposable element inversions [13]. Nevertheless, such inversions have barely been documented. Examples of transposable element inversions include the case of a eukaryotic Tc1/mariner element inserted in a baculovirus after experimental virus infection of the insect host [14], and inversions of the bacterial Tn5 transposon in *Escherichia coli* and various viruses [15]–[17]. While the eukaryotic Tc1/mariner inversion indeed results from ectopic recombination between TIRs, the bacterial Tn5 inversions are not TIR-TIR recombination events *per se*, but rather recombination events between the IS50 insertion sequence elements that flank Tn5 composite transposons [18]. In this study, we report natural inversions generated by TIR-TIR recombination in the most common prokaryotic DNA transposons: insertion sequences (IS) [9], [10].

## Methods

We analyzed an IS element of the IS5 family known as ISWpi1, characterized in bacteria of the genus *Wolbachia* [19]. Multiple ISWpi1 copies occur at various genomic sites in various *Wolbachia* genomes [19]–[21]. Sequence data from 22 ISWpi1 insertion loci amplified and sequenced from multiple *Wolbachia* strains with known phylogenetic relationships were obtained from a previous study [20]. The sequences used in this study are available in GenBank under accession numbers EU714507–EU714683 [20]. In addition, for the *w*Mel#2 and *w*Mel#7 loci, BLASTn searches were performed against various *Wolbachia* genome sequences available in GenBank, to extend the sequence datasets [22]–[26]. Sequences were aligned using ClustalW as implemented in the software BioEdit ver. 7.0 [27], followed by manual adjustments. ISWpi1 TIRs and transposase open reading frames, as well as flanking direct repeats generated upon insertion were identified following [19]. Transcription initiation motifs were searched manually. They included the -35 and -10 promoter regions, with consensus sequences T*T*G*ACA and T*A*TAAT*, respectively, where positions with an asterisk are the most conserved [28].

## Results

**Figure 1:**
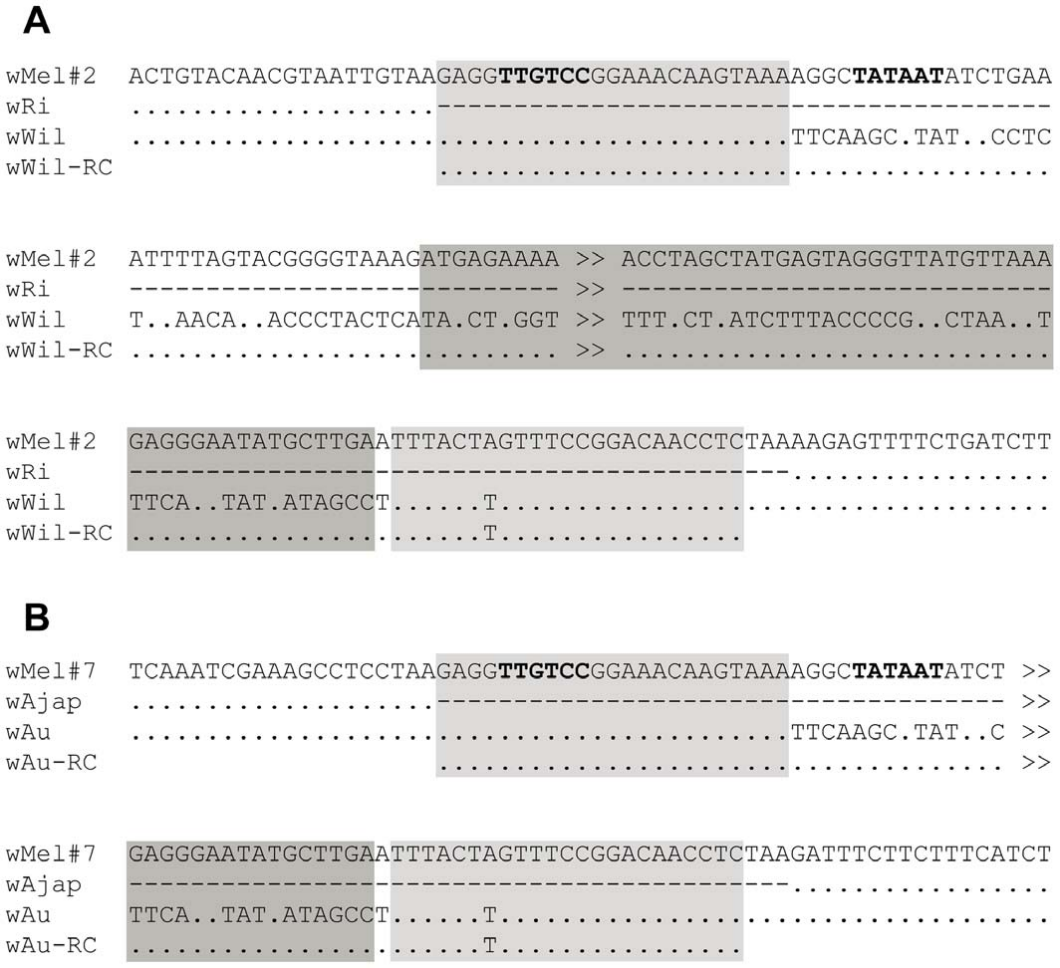
Nucleotide sequence alignments of the ISWpi1 loci *w*Mel#2 (a) and *w*Mel#7 (b). For each locus, four sequences are shown, from top to bottom: the *w*Mel genome sequence displaying a canonical ISWpi1 insertion, a strain which lacks the ISWpi1 insertion, a strain with an inverted ISWpi1 sequence and the reverse-complement sequence of the above inverted ISWpi1 element. ISWpi1 terminal inverted repeats are shown as light-grey boxes and transposase genes as dark-grey boxes. Putative -10 and -35 boxes are bolded. Direct repeats flanking ISWpi1 insertions are boxed. Symbols: -, sequence gap; ., nucleotide identical to top sequence; >>, a portion of the sequence is not shown.

Inspection of the sequence alignments from 22 ISWpi1 loci revealed two loci displaying unusual IS sequence structure. The two ISWpi1 loci, named *w*Mel#2 and *w*Mel#7, were located at coordinates 126,231–127,146 and 532,256–533,171 relative to the *w*Mel *Wolbachia* genome, respectively [19]. The ISWpi1 insertion was inverted in two *Wolbachia* strains at the *w*Mel#2 locus and in one *Wolbachia* strain at the *w*Mel#7 locus, relative to the other strains (Fig. 1).

**Figure 2:**
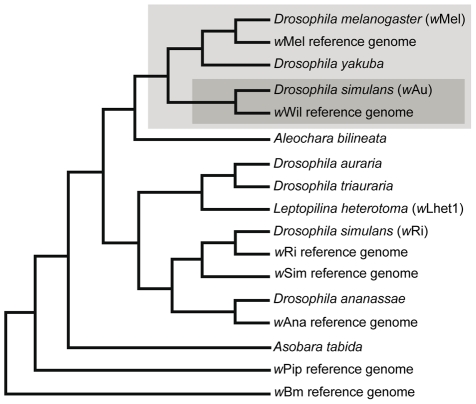
Evolutionary history of ISWpi1 insertion and inversion at the *w*Mel#2 locus. The phylogenetic tree of *Wolbachia* strain relationships is from [20]. *Wolbachia* strains are identified by the name of the host species from which they were isolated, except for the published genome sequences (“reference genomes”). *Wolbachia* strains with the ISWpi1 insertion at the *w*Mel#2 locus are shown in a light-grey box and strains with the inverted ISWpi1 insertion are shown in a dark-grey box. All other strains lack the ISWpi1 insertion.

To clarify the evolutionary history of the inversion pattern found in the two *Wolbachia* strains at the *w*Mel#2 locus, we mapped the distribution of ISWpi1 insertion and inversion patterns onto a phylogeny of *Wolbachia* strains (Fig. 2). The most parsimonious interpretation of this analysis was that a single canonical ISWpi1 insertion event occurred in the ancestor of a monophyletic group of closely related *Wolbachia* strains (Fig. 2, light gray box). Subsequently, a single ISWpi1 inversion event occurred in the ancestor of the two highly closely related *w*Wil and *w*Au *Wolbachia* strains (Fig. 2, dark gray box).

**Figure 3:**
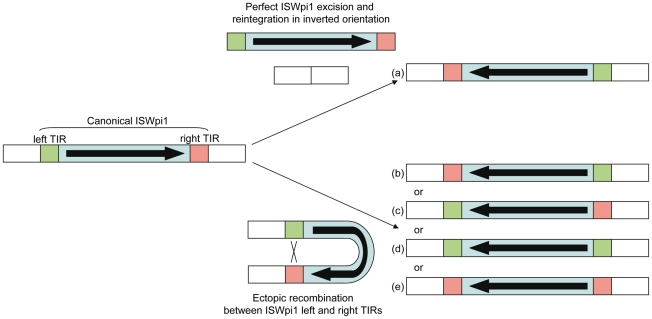
Predictions of the different potential mechanisms causing ISWpi1 inversions. A canonical ISWpi1 insertion is shown on the left, with the left and right terminal inverted repeats (TIR) shown in green and red, respectively. The transposase gene is shown in blue and the thick arrow indicates its orientation. The five predicted outcomes of the inversion process (a–e) are shown on the right.

The question arises as to what mechanism may be responsible for this inverted pattern. As ISWpi1 contains a potentially functional transposase gene, has experienced recent transpositional activity in *Wolbachia* and targets genomic insertion sites with sequence T(T/A)A [19], [20], it is plausible that the ISWpi1 copies excised from the *w*Mel#2 and *w*Mel#7 loci and reintegrated again at the exact same insertion sites in inverted orientation (Fig. 3). Alternatively, ectopic recombination between the TIRs that bound ISWpi1 elements may have caused the inversion of the intervening sequences (Fig. 3).

These hypotheses can be tested because they make different predictions regarding the sequence configuration of the asymmetric ISWpi1 TIRs, which are identical 23 bp-long sequences except for a single A/T nucleotide mismatch corresponding to nucleotide position (np) 17 in the left TIR [19]. Indeed, ISWpi1 excision and reintegration is expected to lead to the inversion of the entire element, including the transposase gene and the TIRs (Fig. 3a). By contrast, ectopic recombination between TIRs is expected to produce different outcomes depending on the location in the TIRs of the conversion tract allowing the formation of the recombining heteroduplex [29]–[31]:

- The conversion tract is located upstream of the nucleotide mismatch of the TIRs, i.e. between np 1 and 16: ectopic recombination results in the inversion of the transposase gene along with the nucleotide mismatch at np 17, thereby leading to the inversion of the TIRs (Fig. 3b);
- The conversion tract is located downstream of the nucleotide mismatch of the TIR, i.e. between np 18 and 23: ectopic recombination results in the inversion of the transposase gene only. Because the nucleotide mismatch at np 17 is not inverted in the process, the TIRs are not inverted either (Fig. 3c);
- The conversion tract encompasses nucleotide mismatch at np 17: ectopic recombination results in the inversion of the transposase gene and the nucleotide mismatch at np 17 is lost, leading to TIRs with identical sequences. If the left TIR is used as template for gene conversion, then the right TIR becomes identical to the left TIR (Fig. 3d). Alternatively, if the right TIR is used as template for gene conversion, then the left TIR becomes identical to the right TIR (Fig. 3e).

Our data indicate that the left and right TIRs of the inverted IS elements are identical to each other at both the *w*Mel#2 and *w*Mel#7 loci (see *w*Wil and *w*Wil-RC alignment in Fig. 1a, and *w*Au and *w*Au-RC alignment in Fig 1b), thereby invalidating scenarios (a), (b) and (c) shown in Fig. 3. In addition, at both loci, the left TIRs of the inverted IS elements are identical to the canonical ISWpi1 left TIR, but not to the canonical right TIR (see *w*Mel#2 and *w*Wil alignment Fig. 1a, and *w*Mel#7 and *w*Au alignment in Fig 1b). Thus, the observed ISWpi1 inversion patterns at the *w*Mel#2 and *w*Mel#7 loci correspond to configuration (d) shown in Fig 3. We conclude that the ISWpi1 inversion events at the *w*Mel#2 and *w*Mel#7 loci were generated by ectopic recombination between the TIRs of ISWpi1 copies, with concomitant conversion of the right TIR sequence into that of the left TIR.

## Discussion

To our knowledge, this is the first report of TIR-TIR recombination within bacterial IS elements. ISWpi1 inversion events were isolated from natural *Wolbachia* bacterial strains [20]. This indicates that TIR-TIR recombination occurs naturally in an evolutionary context, and is not the result of particular conditions of laboratory experiments. It is difficult to assess whether or not this phenomenon is widespread in prokaryotes. This is because the characterization of an IS inversion requires a reliable sequence alignment from many bacterial strains encompassing both the IS insertion and its flanking genomic sequences (which anchor the orientation of the sequences under investigation). Unfortunately, IS annotation is rarely optimal in completely sequenced prokaryotic genomes available at this time and, for many prokaryotic species, only one or few genomes have been sequenced. Thus, it is presently difficult to characterize and quantify the significance of TIR-TIR recombination and resulting IS inversions on prokaryote genomic plasticity and evolution. Yet, we identified IS inversions in 9% (2/22) of ISWpi1 loci investigated in *Wolbachia*. This suggests that TIR-TIR recombination may be a quite common, and yet largely overlooked, source of genomic diversity in bacteria. The rapid development of next-generation sequencing approaches coupled with improved IS annotation procedures will soon provide the opportunity to investigate such IS inversions on a large scale. In any event, our results provide proof of concept that ectopic recombination between TIRs can mediate IS inversions. They also contribute to extend our understanding of the emerging complexity of IS element structures, which vary from solo-TIRs to more complex IS elements that can carry passenger genes [10]–[12], [32].

The question arises as to what may be the consequences of such inversions on IS function. In the two cases we characterized, the asymmetry between the TIRs was abolished as a result of the recombination process. Generally, IS transcription is tightly regulated and endogenous transcriptional promoters are often partially located in the TIRs [9], [33]. In canonical ISWpi1 elements, the putative -10 box (TATAAT) is located downstream of the left TIR, at np 28–33 relative to the beginning of the left TIR (Fig. 1). Therefore, in inverted ISWpi1 elements, the -10 box gets inverted along with the transposase gene, thus preserving the original configuration. By contrast, the putative -35 box (TTGTCC), which shows the typical 17-bp spacing with the -10 box [28], is located inside the left TIR, at np 5–10 relative to the beginning of the left TIR (Fig. 1). However, because the left TIR sequence is found at both TIRs of inverted ISWpi1 elements, the -10 box is also in an appropriate configuration in inverted elements. Overall, the TIR-TIR recombination process has resulted in the appropriate repositioning of both transcriptional promoters relative to the inverted transposase gene. Thus, we conclude that transcription of the transposase gene most likely is not impaired in inverted ISWpi1 elements.

TIRs of IS elements are also crucial for transposition in that they usually contain transposase binding sites and asymmetry in the TIRs may serve to distinguish the left and right TIRs during the excision process [9], [33]. As TIR asymmetry has been lost in inverted ISWpi1 elements, it is possible that transposition efficiency is altered in inverted elements compared to canonical elements.

**Figure 4:**
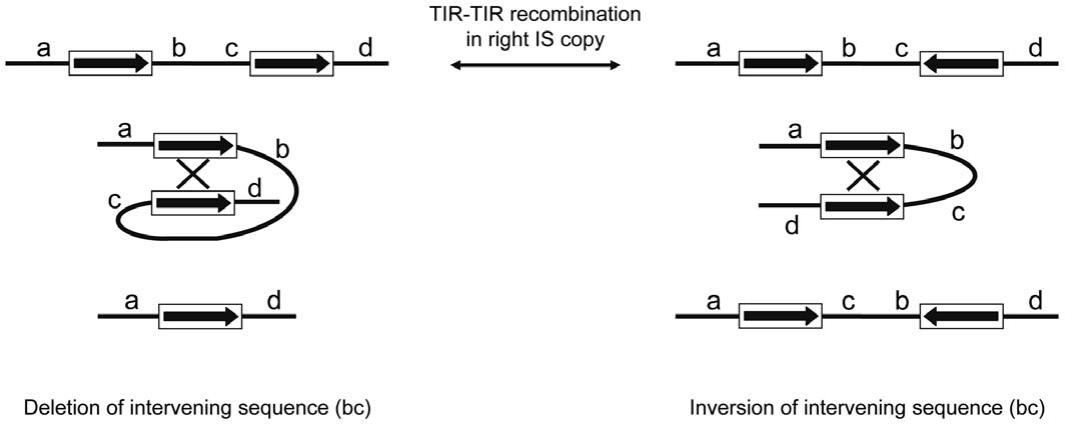
Genomic rearrangements generated by ectopic recombination between IS copies according to their relative orientation. Ectopic recombination between IS copies in the same (left) or opposite (right) relative orientation is shown. Copies are shown as white boxes with internal arrows representing their orientation. Thick black lines represent flanking genomic sequences (a–d regions). TIR-TIR recombination within an IS copy will change the relative orientation of two neighbouring copies, which will alter the outcome of a subsequent recombination event between the two copies.

Another potential implication of IS inversions relates to genomic instability. Indeed, the occurrence of multiple homologous transposable element sequences within genomes makes them prone to ectopic recombination, which may result in genomic rearrangements such as deletions and inversions. Transposable element-induced rearrangements have been reported in many bacterial genomes [34], [35], including *Wolbachia* [26], [36]. Importantly, the relative orientation of two recombining transposable elements determines the outcome of the recombination process. Indeed, recombination between copies in the same orientation is expected to result in the deletion of the intervening sequence (Fig. 4, left) whereas recombination between copies in opposite orientations is expected to result in the inversion of the intervening sequence (Fig. 4, right). Thus, recombination between TIRs of a given IS copy resulting in its inversion is expected to alter the outcome of ectopic recombination events that would subsequently involve such inverted IS elements and other homologous elements (Fig. 4). While genomic inversions may have relatively mild consequences on host fitness, TIR-TIR recombination-mediated IS inversions may “trigger” potentially more deleterious genomic instability such as genomic deletions. Conversely, IS inversions may be selected to decrease the deleteriousness of neighbouring IS elements originally inserted in the same orientation (Fig. 4). If so, IS inversions may constitute a potentially important regulator of IS-induced genomic rearrangements and instability.

In summary, we provided evidence for recombination between the TIRs of IS copies, which uncovers a novel mechanism of structural variation for this type of prokaryotic transposable elements. Interestingly, our results suggest that recombination events required very short regions of homology, as ISWpi1 TIR is only 23 bp in length [19]. We identified IS inversions in nearly one tenth of all ISWpi1 loci investigated in *Wolbachia*, which suggests that TIR-TIR recombination may be a quite common, albeit largely overlooked, source of genomic diversity in bacteria. IS inversions may impede transposable element survival and expansion in the host genome by altering transpositional activity. They may also affect genomic instability by modulating the outcome of ectopic recombination events between IS copies in various orientations.

## References

[1] Cordaux R, Batzer MA (2009). The impact of retrotransposons on human genome evolution.. Nat Rev Genet.

[2] Treangen TJ, Abraham AL, Touchon M, Rocha EP (2009). Genesis, effects and fates of repeats in prokaryotic genomes.. FEMS Microbiol Rev.

[3] Hedges DJ, Deininger PL (2007). Inviting instability: Transposable elements, double-strand breaks, and the maintenance of genome integrity.. Mutat Res.

[4] Wicker T, Sabot F, Hua-Van A, Bennetzen JL, Capy P (2007). A unified classification system for eukaryotic transposable elements.. Nat Rev Genet.

[5] Belshaw R, Watson J, Katzourakis A, Howe A, Woolven-Allen J (2007). Rate of recombinational deletion among human endogenous retroviruses.. J Virol.

[6] Lander ES, Linton LM, Birren B, Nusbaum C, Zody MC (2001). Initial sequencing and analysis of the human genome.. Nature.

[7] Vitte C, Panaud O (2003). Formation of solo-LTRs through unequal homologous recombination counterbalances amplifications of LTR retrotransposons in rice Oryza sativa L.. Mol Biol Evol.

[8] Feschotte C, Pritham EJ (2007). DNA transposons and the evolution of eukaryotic genomes.. Annu Rev Genet.

[9] Chandler M, Mahillon J, Craig NL, Craigie R, Gellert M, Lambowitz AM (2002). Insertion sequences revisited.. Mobile DNA II.

[10] Siguier P, Filee J, Chandler M (2006). Insertion sequences in prokaryotic genomes.. Curr Opin Microbiol.

[11] Filee J, Siguier P, Chandler M (2007). Insertion sequence diversity in archaea.. Microbiol Mol Biol Rev.

[12] Pichon S, Bouchon D, Cordaux R, Chen L, Garrett RA (2009). Conservation of the Type IV secretion system throughout Wolbachia evolution.. Biochem Biophys Res Commun.

[13] Andersson SG, Kurland CG (1998). Reductive evolution of resident genomes.. Trends Microbiol.

[14] Arends HM, Jehle JA (2002). Homologous recombination between the inverted terminal repeats of defective transposon TCp3.2 causes an inversion in the genome of Cydia pomonella granulovirus.. J Gen Virol.

[15] Weber PC, Challberg MD, Nelson NJ, Levine M, Glorioso JC (1988). Inversion events in the HSV-1 genome are directly mediated by the viral DNA replication machinery and lack sequence specificity.. Cell.

[16] Weber PC, Levine M, Glorioso JC (1988). Simple assay for quantitation of Tn5 inversion events in Escherichia coli and use of the assay in determination of plasmid copy number.. J Bacteriol.

[17] Martin DW, Weber PC (1997). DNA replication promotes high-frequency homologous recombination during Autographa californica multiple nuclear polyhedrosis virus infection.. Virology.

[18] Reznikoff WS, Craig NL, Craigie R, Gellert M, Lambowitz AM (2002). Tn5 transposition.. Mobile DNA II.

[19] Cordaux R (2008). ISWpi1 from Wolbachia pipientis defines a novel group of insertion sequences within the IS5 family.. Gene.

[20] Cordaux R, Pichon S, Ling A, Perez P, Delaunay C (2008). Intense transpositional activity of insertion sequences in an ancient obligate endosymbiont.. Mol Biol Evol.

[21] Cordaux R (2009). Gene conversion maintains nonfunctional transposable elements in an obligate mutualistic endosymbiont.. Mol Biol Evol.

[22] Wu M, Sun LV, Vamathevan J, Riegler M, Deboy R (2004). Phylogenomics of the reproductive parasite Wolbachia pipientis wMel: a streamlined genome overrun by mobile genetic elements.. PLoS Biol.

[23] Foster J, Ganatra M, Kamal I, Ware J, Makarova K (2005). The Wolbachia genome of Brugia malayi: endosymbiont evolution within a human pathogenic nematode.. PLoS Biol.

[24] Salzberg SL, Hotopp JC, Delcher AL, Pop M, Smith DR (2005). Serendipitous discovery of Wolbachia genomes in multiple Drosophila species.. Genome Biol.

[25] Klasson L, Walker T, Sebaihia M, Sanders MJ, Quail MA (2008). Genome evolution of Wolbachia strain wPip from the Culex pipiens group.. Mol Biol Evol.

[26] Klasson L, Westberg J, Sapountzis P, Naslund K, Lutnaes Y (2009). The mosaic genome structure of the Wolbachia wRi strain infecting Drosophila simulans.. Proc Natl Acad Sci U S A.

[27] Hall TA (1999). BioEdit: a user-friendly biological sequence alignment editor and analysis program for Windows 95/98/NT.. Nucl Acids Symp Ser.

[28] Rosenberg M, Court D (1979). Regulatory sequences involved in the promotion and termination of RNA transcription.. Annu Rev Genet.

[29] Marais G (2003). Biased gene conversion: implications for genome and sex evolution.. Trends Genet.

[30] Santoyo G, Romero D (2005). Gene conversion and concerted evolution in bacterial genomes.. FEMS Microbiol Rev.

[31] Barzel A, Kupiec M (2008). Finding a match: how do homologous sequences get together for recombination?. Nat Rev Genet.

[32] Siguier P, Gagnevin L, Chandler M (2009). The new IS1595 family, its relation to IS1 and the frontier between insertion sequences and transposons.. Res Microbiol.

[33] Nagy Z, Chandler M (2004). Regulation of transposition in bacteria.. Res Microbiol.

[34] Parkhill J, Sebaihia M, Preston A, Murphy LD, Thomson N (2003). Comparative analysis of the genome sequences of Bordetella pertussis, Bordetella parapertussis and Bordetella bronchiseptica.. Nat Genet.

[35] Yang F, Yang J, Zhang X, Chen L, Jiang Y (2005). Genome dynamics and diversity of Shigella species, the etiologic agents of bacillary dysentery.. Nucleic Acids Res.

[36] Leclercq S, Giraud I, Cordaux R (2010). Remarkable Abundance and Evolution of Mobile group II Introns in Wolbachia Bacterial Endosymbionts.. Mol Biol Evol.

